# An Additively Optimal Interpreter for Approximating Kolmogorov Prefix Complexity

**DOI:** 10.3390/e26090802

**Published:** 2024-09-20

**Authors:** Zoe Leyva-Acosta, Eduardo Acuña Yeomans, Francisco Hernandez-Quiroz

**Affiliations:** 1 Ciencia e Ingenieria de la Computacion, Universidad Nacional Autónoma de México, Ciudad Universitaria, Mexico City 04510, Mexico; zoeleyva18@gmail.com; 2 Facultad de Ciencias, Universidad Nacional Autónoma de México, Ciudad Universitaria, Mexico City 04510, Mexico; fhq@ciencias.unam.mx

**Keywords:** algorithmic complexity, Kolmogorov complexity, coding theorem method, complexity approximation

## Abstract

We study practical approximations of Kolmogorov prefix complexity (*K*) using IMP2, a high-level programming language. Our focus is on investigating the optimality of the interpreter for this language as the reference machine for the Coding Theorem Method (CTM). This method is designed to address applications of algorithmic complexity that differ from the popular traditional lossless compression approach based on the principles of algorithmic probability. The chosen model of computation is proven to be suitable for this task, and a comparison to other models and methods is conducted. Our findings show that CTM approximations using our model do not always correlate with the results from lower-level models of computation. This suggests that some models may require a larger program space to converge to Levin’s universal distribution. Furthermore, we compare the CTM with an upper bound on Kolmogorov complexity and find a strong correlation, supporting the CTM’s validity as an approximation method with finer-grade resolution of *K*.

## 1. Introduction

The algorithmic Kolmogorov complexity of a string is the length of the smallest program that produces the string when executed by a reference machine [1]. Apart from being an uncomputable measure, another inconvenience of algorithmic complexity is that, in practice, the choice of the reference model or description method plays a role in approximating calculations. In that sense, the Invariance Theorem [2] tells us about the existence of some optimal universal machines such that, when chosen as the reference model, they produce an invariant measure, up to an additive constant. This constant can skew the Kolmogorov complexity values of short strings, as the invariance, even in the optimal theoretical case, is of an asymptotic nature.

An approach to algorithmic complexity introduced previously [3,4] is based on the so-called *Coding Theorem Method* (CTM) [3,5,6,7], which relies on a relation between the algorithmic prefix complexity of a string and its algorithmic probability [2,8]. The method approximates the value of a string’s algorithmic complexity via the frequency distribution of that string as an output of small Turing machines and other models. Zenil and Delahaye [9] pioneered this empirical approach to algorithmic complexity based on output frequency distributions across different computational models (e.g., Turing machines, cellular automata, and Post systems) as well as empirical distributions from real-world/physical data.

A criticism of the CTM [10] is its potential dependency on the reference machine, which may affect the stability of the method when the reference model changes. The only other alternative, based on popular lossless compression, does not address this problem either and is more related to classical Information Theory (Shannon entropy) than to Kolmogorov complexity [11]. The findings of Zenil et al. [9] revealed a tendency towards simplicity occurring with greater frequency and some alignment across models. As the CTM depends on the outputs from the execution of a subset of programs for a particular model of computation, changing the subset of programs or the reference model could lead to inconsistent results, even when in compliance with the theory. However, the theory guarantees convergence, and in this work, we explore how slowly or quickly a higher-level programming language may converge.

The authors of the CTM have reported empirical evidence in favour of the stability of complexity approximations despite changes in the computational formalism when applying the CTM [9,11,12,13], further suggesting a convergence towards some early natural distribution based on the complexity of the underlying model of computation [14], even without the guarantee of optimality. The conjecture in [14] can be summarised as implying that the simpler (shorter) a model of computation, the faster it may converge. The early reported correspondence between high algorithmic probability strings from output frequency distributions has been suggested as a defining characteristic of ‘natural’ computational models [14], specifically those aligning with the distributions observed in Turing machines and cellular automata. This led to the conjecture that simpler models converge faster than more complex or artificial ones.

In this paper, we introduce a computational model that aspires to meet both desirable theoretical constraints and practical applicability. To begin with, the model is optimal. Additionally, it is based on a high-level programming language, which, ideally, can offer shorter versions of program routines performing abstract tasks. The optimal universal interpreter for our language, called IMP2, is constructed in such a way that a well-defined Kolmogorov prefix complexity is specified. This entails both a suitable choice for a binary prefix-free encoding of programs and an efficient enumeration of programs. Moreover, given that our proposed model conforms to the theory of algorithmic prefix complexity, it can be used for independent empirical tests of the previously suggested hypothesis regarding the convergence towards a ‘natural’ distribution upon application of the CTM.

The computational methods implemented allow us to generate all IMP2 programs up to a given program length, as well as execute each program in a resource-bounded evaluator in order to obtain the output strings. Our framework allows us to produce approximations to the Kolmogorov complexity of a relatively small set of strings, using both the CTM and a more direct approximation based on the length of the smallest program found in the program space, which we call SPF. This was achieved with very modest computational means, just to get a taste of what can be done with the language before embarking on larger-scale calculations.

Our results show that the degree of correlation between the estimated CTM complexity under IMP2 and the previous estimation values published in the literature [5,7] varies widely and significantly, depending on whether all output strings are considered or only those of the same length. The differences we found in complexity estimations under IMP2 and other models of computation indicate that some models might not conform to the algorithmic ‘natural’ behaviour observed previously [9,11,12]. This further suggests that the methodology of the CTM is, in fact, sensitive to changes in the reference model chosen, even if such a model is not biased towards being non-optimal or unreasonable. However, surprisingly, we also found (a) a correlation between the approximations via the CTM and the length of the smallest program found (SPF) for each string under our own reference machine and (b) that the SPF is a coarse-grained measurement of the CTM. These two facts further support the claims of the validity of the CTM as an approach to algorithmic complexity presented in previous reports [15], even if the empirical approximations produced are not exempt from the theoretical drawbacks arising from dependence on the chosen model.

## 2. Preliminaries

The *algorithmic complexity* [1], also known as *Kolmogorov complexity*, of a string of bits *x* with respect to a universal Turing machine *U* is defined as

$$K_{U}\left(x\right)=\min\left\{|p|\;:\;U(p)=x\right\},$$

where |p| denotes the length in bits of the program *p* and U(p)=x means that the universal Turing machine *U*, given input *p*, produces output *x* upon completing its execution. On the other hand, *prefix complexity* is a variant of plain Kolmogorov complexity, as defined above, where we require the reference Turing machine *U* to be *prefix-free*, that is, the set of programs it can execute forms a prefix-free set (meaning no program is a prefix of another).

A machine *U* is *additively optimal* [2] for a class of machines (such as the class of Turing machines or prefix machines), if for any other machine $U^{\prime }$ of the same class, there is a constant *c* (depending on *U* and $U^{\prime }$ but not on *x*) such that for all *x*,

(1) $$K_{U}\left(x\right)\leq K_{U^{\prime }}\left(x\right)+c$$

Moreover, the Invariance Theorem guarantees the existence of additively optimal machines for both Turing machines and prefix machine classes. In what follows, for simplicity, we refer to additively optimal machines as optimal.

The *algorithmic probability* (also known as the universal distribution) [16] of a string *x* with respect to a Turing machine *U* is defined as

$$m_{U}\left(x\right)=\sum_{U(p)=x}2^{-|p|}$$

The CTM [5] aims to produce an empirical approximation of algorithmic probability by obtaining the output strings from the execution of all Turing machines with *n* states and *m* symbols, i.e., the set (n,m). Then, the relative frequency, D(n,m)(x), of the output string *x* with respect to the total number of halting machines in (n,m) is taken as an approximation of its algorithmic probability:

(2) $$D\left(n,m\right)\left(x\right)=\frac{|\left\{p\in \left[1,|\left(n,m\right)|\right]:T_{p}\left(\epsilon \right)=x\right\}|}{|\left\{p\in \left[1,|\left(n,m\right)|\right]:T_{p}\mathrm{halts}\right\}|},$$

where $T_{p}$ denotes the Turing machine with number *p* in the set (n,m), according to the enumeration in [5].

The relationship between algorithmic probability and prefix Kolmogorov complexity is established by the *Coding Theorem* [2], which states that there is a constant *c* such that for every string *x*,

$$|-\log_{2}m_{U}\left(x\right)-K_{U}\left(x\right)|<c.$$

Based on this relation, given that the constant bound is asymptotically non-significant, the CTM approximates the complexity of a string *x* by

(3) $$CTM_{\left(n,m\right)}\left(x\right)=-\log_{2}D_{\left(n,m\right)}\left(x\right).$$

## 3. Methodology and Techniques

One of the main goals of our study was to obtain a reference computational model that meets some desirable constraints, specifically being optimal and prefix-free. We call this reference machine IMP2, as it is the result of major modifications to IMP, a very well-known, small imperative language used in [17], with the purpose of achieving provable prefix-free optimality.

In order to adapt the CTM to our IMP2, we enumerate its programs by increasing their length, and then we execute them on a resource-bounded interpreter. When the number of execution steps exceeds a given threshold [17], the program is considered non-halting. When a program halts, an output string is computed, and the relative frequency of the output is adjusted accordingly. The frequency values for each output string are then used to obtain the CTM approximation of algorithmic complexity under the IMP2 model.

Additionally, the length of the first program producing a string is also registered as its SPF (*smallest program found*) complexity approximation. Of course, the unsolvability of the halting problem prevents considering the SPF’s length as the Kolmogorov complexity of the string under IMP2.

### 3.1. From IMP to IMP2

In [17], the authors presented a proof of concept for an approach to approximate plain Kolmogorov complexity using the SPF, based on a reference machine called IMP. Although this is a reasonable computational model based on an imperative high-level programming language, proving whether this model is optimal is not straightforward. The same is also true for other similar attempts to approximate Kolmogorov prefix complexity [5,7], where the approaches are based on a reference universal Turing machine with a reasonable or ‘natural’ design, whose optimality is also merely conjectured.

As for the prefix-free property, since the Coding Theorem is central to the CTM, one of the approximation methods we focus on in this study, we require our chosen reference machine to be prefix-free. However, it is difficult to consider IMP, as used in the approach presented in [17], a suitable reference model for a well-specified prefix Kolmogorov complexity. This is mainly because the approach to ‘program length’ used in [17] is associated with the number of nodes in the abstract syntax tree of the corresponding sentence of the high-level programming language grammar. Even though a prefix-free encoding could be devised for IMP language programs, there is no well-defined binary encoding of ‘programs’ in the approach presented in [17], preventing a direct interpretation of program length as the number of bits in its binary representation. The presence of such binary encoding for the input programs of the reference machine is crucial for this length interpretation, as well as for satisfying the prefix-free property, a fundamental requirement for a well-specified Kolmogorov prefix complexity. The specification of such encoding for the input programs is one of the most significant differences between our current model proposal, IMP2, and the one presented in [17].

The development of the IMP2 model was predominantly driven by the aim of creating a reference machine suitable for approximating Kolmogorov prefix complexity while adhering to the fundamental design principles of the original IMP framework. This led to the creation of a fundamentally distinct model that is suitable for proving optimality and preserves the syntactic structure, output conventions, and semantic properties of the IMP language.

### 3.2. Syntax of IMP2

**Figure 1:**
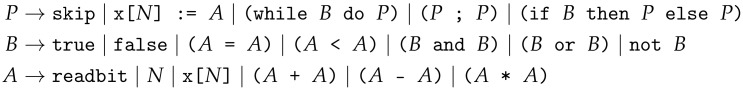
Context-free grammar for IMP2. *N* stands for natural numbers without leading zeros.

IMP2 is a small high-level imperative programming language. The language’s syntax is specified by the context-free grammar shown in Figure 1.

A well-specified input program consists of a two-part code 〈n,y〉, defined as

(4) $$\langle n,y\rangle =\bar{n}\cdot y$$

where *n* specifies a particular sentence $P_{n}$ of IMP2 by its index *n* in an enumeration of all valid sentences of the language (see Section 3.6) and $\bar{n}$ denotes its self-delimiting binary encoding. For the latter, consider the string $B_{n}$ according to the length-increasing lexicographic enumeration of all binary strings:

$$\left(\epsilon ,0\right),\left(0,1\right),\left(1,2\right),\left(00,3\right),\left(01,4\right),\left(10,5\right),\left(11,6\right),\ldots ,\left(B_{n},n\right),\ldots$$

Then, $\bar{n}=1^{|B_{n}|}\cdot 0\cdot B_{n}$ (the prefix $1^{|B_{n}|}\cdot 0$ is for making it self-delimiting). *y* is a binary input stream.

### 3.3. Semantics of IMP2

The semantics are based on an array of memory locations and a potentially empty stream of input bits. The execution of an IMP2 program 〈n,y〉 starts by scanning and parsing the *n*-part of the input program. The second step involves identifying the *n*-th IMP2 sentence $P_{n}$ according to the enumeration presented in Section 3.6. The machine then sets all memory locations to 0 and prepares to read the input stream *y*. The interpreter the executes $P_{n}$ with *y* in the input tape. Commands have the usual meanings as in standard high-level programming languages.

The special arithmetic operation readbit allows programs to read a single bit from the input stream, starting from the first bit of the string *y*; this is the only way to scan the input. The next readbit operation reads the following bit, meaning the bits are read in order and cannot be read twice. If the program reads from an empty input stream, it loops indefinitely and is thus considered a non-halting program. An assignment of readbit to a memory location stores the bit read in that location.

**Figure 2:**
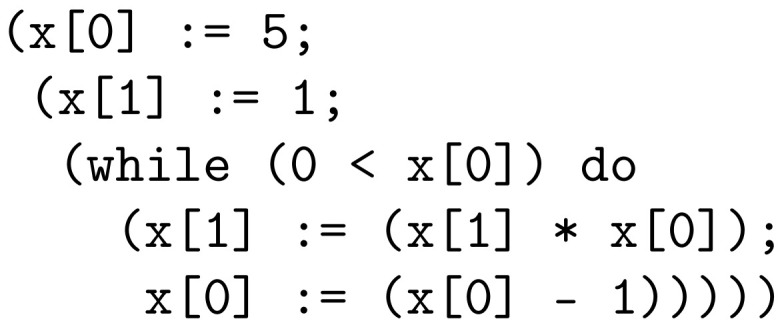
Example of an IMP2 sentence computing 5! and storing the result in location 1.

**Figure 3:**
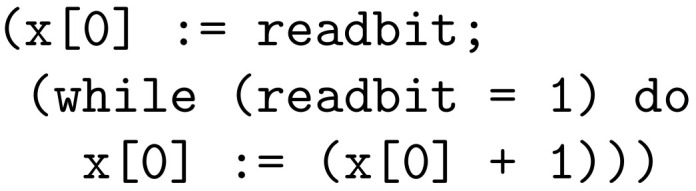
Example of an IMP2 sentence counting consecutive 1 bits from the input stream and storing the result in location 0.

Memory locations are indexed by non-negative integers and contain arbitrarily large non-negative integers. Figure 2 and Figure 3 show examples of IMP2 sentences.

A statistical algorithm, based on sampling the program space to estimate a halting threshold [18], was incorporated into the IMP2 interpreter to ensure termination and mitigate the effects of the halting problem [17].

IMP2 has all the necessary features to compute any partial computable function and, therefore, it is Turing complete.

### 3.4. Input/Output Convention

A valid halting IMP2 program 〈n,y〉 is one where the machine halts after reading exactly all the bits from the input stream. For example, if the execution of the program 〈n,001〉 halts after invoking the readbit operation only twice, this means that 〈n,001〉 is not a valid IMP2 program, whereas 〈n,00〉 is.

The result of the execution of a halting program is determined by the state of the memory locations. To be useful for approximating algorithmic complexity, the contents of the memory locations must be interpreted as a particular binary string. Although many interpretations are possible, the chosen one is the concatenation of the binary strings corresponding to the values in each of the memory locations of a program. The conversion of non-negative integers to binary strings follows the previous convention, and the concatenation of strings is performed in increasing order of the subscripts of the memory locations.

As an example, consider the sample program for computing 5! in Figure 2. After the program halts, the values stored in memory locations are x[0]=0 and x[1]=120, with other locations containing their initial values of 0, which are mapped to the empty string ϵ. Thus, since $B_{x}\left[0\right]=\epsilon$ and $B_{x}\left[1\right]=111001$, the resulting output string is ϵ·111001=111001.

Thus, we say that IMP2 produces the string *x* as *output*, with *input*〈n,y〉, and write $\mathrm{IMP}2\left(\langle n,y\rangle \right)=P_{n}\left(y\right)=x$, if the segment of the input scanned at the moment of halting is exactly the string 〈n,y〉, and $x=B_{x}\left[0\right]\cdot B_{x}\left[1\right]\ldots$.

### 3.5. Prefix-Free Optimality

Prefix-free machines, or simply prefix machines, are also known in the literature as *self-delimiting* machines [2,19]. Under our convention for inputs and outputs, it is not difficult to see that IMP2 is a universal prefix machine. Notice that the two-part code 〈n,y〉 of an IMP2 program uses the previously defined self-delimiting encoding $\bar{n}$. Moreover, IMP2 is designed to accept an input program on its own, without using an end-of-string indication to stop scanning the input tape. This ensures that the set of strings accepted, i.e., the set of halting programs, is prefix-free.

**Proposition** **1.** *The universal machine IMP2 is additively optimal for the class of prefix machines.*

**Proof.**  Consider an arbitrary prefix machine *G* over a binary input alphabet. Since IMP2 is a universal machine, there exists an IMP2 sentence $P_{n}$, such that, for any binary string *y*,

$$\mathrm{IMP}2\left(\langle n,y\rangle \right)=P_{n}\left(y\right)=G\left(y\right)$$

Hence, considering that $\bar{n}=1^{|B_{n}|}\cdot 0\cdot B_{n}$ and $|\bar{n}\left|=2(\right|B_{n}\left|\right)+1$, it follows from the definition of the algorithmic complexities $K_{\mathrm{IMP}2}$ and $K_{G}$ that

$$K_{\mathrm{IMP}2}\left(x\right)\leq K_{G}\left(x\right)+2(|B_{n}\left|\right)+1,$$

where $2(|B_{n}|)+1$ is a constant depending only on IMP2 and *G*. Therefore, it follows that IMP2 is an additively optimal machine for the class of prefix machines. □

### 3.6. Enumeration of IMP2 Sentences

Sentences of the IMP2 language are enumerated based on the structure of syntactically valid abstract syntax trees (ASTs). Using standard methods for enumerating combinatorial structures [20,21], we implemented a system for constructing enumerators based on combinators [17,22] that allow us to build and compose complex enumerations from simpler ones. The enumerations of numbers, truth values, and other atomic elements are combined to produce enumerations of arithmetic and boolean expressions, which are themselves combined to produce enumerations of assignments and control structures, all the way up to the top-level sentences specified by the language grammar.

The bijection between sentences and natural numbers is encoded with a pair of functions. The rank function produces the unique position of a given AST, and the unrank function is the inverse, producing a unique AST given its position.

The most important method of combination consists of obtaining the product of two or more enumerations, where the positions are interweaved using Cantor’s pairing function in the binary case and a generalisation for *n*-tuples of natural numbers, which satisfies the fairness property [22].

When combining enumerations for a particular syntactic category, such as arithmetic expressions, boolean expressions, or IMP2 sentences, the positions are partitioned in an alternating fashion between the production rules.

Considering the program for calculating 5! in Figure 2 and the enumeration of IMP2 sentences, the assignment x[0]:=5 is mapped to position 1405, the more complex assignment x[1]:=(x[1]∗x[0]) is mapped to position 142049, the while loop in the example is mapped to position 17972673899864641600766, and the whole example sentence is mapped to a position whose decimal representation contains 90 digits.

### 3.7. Enumerating IMP2 Programs by Length

The enumeration of programs consisting of a sentence and its input stream must take into account the number of bits in the corresponding two-part code 〈n,y〉 in order to produce a length-increasing order.

For a given code length *m*, we consider all possible combinations of *k*-bit self-delimiting strings $\bar{n}$ and (m−k)-bit binary strings *y* for 0≤k≤m. This can be easily accomplished given the simple structure of the encoding, yielding $2^{m-k}$ binary strings for the input stream and $2^{(k-1)/2}$ sentences when *k* is an odd number, and zero programs otherwise.

The subset of programs of a particular length can be partitioned and enumerated independently, allowing for a distributed enumeration and execution of programs.

## 4. Results

Our preliminary experiment was performed by executing all IMP2 programs of at most length 40, denoted as ${\mathrm{IMP}2}_{40}$. Running this experiment took 34 min in total for estimating the halting threshold and 51.7 h for the enumeration and execution of ${\mathrm{IMP}2}_{40}$. We used a desktop computer, dedicating 30 threads of an AMD Ryzen 9 CPU running at 4.5 GHz with 6 GiB of memory each.

We recorded the length of the smallest program found for every output string *x*, denoted as ${\mathrm{SPF}}_{40}\left(x\right)$. Additionally, for approximating the complexity of strings via the CTM, we recorded the relative frequency of programs producing every output string *x* with respect to the total number of halting programs, denoted as $D\left({\mathrm{IMP}2}_{40}\right)\left(x\right)$. The full dataset containing the complexity estimations can be found at https://kolm-complexity.gitlab.io/optimal-interpreter/ (accessed on 17 September 2024).

To put the scale of our experiment into perspective, we used the empirical frequency distributions D(4,2) and D(5,2) as references, as published in [5,7]. Table 1 shows a summary contrasting some key aspects of the experiments. In the table, the entry corresponding to ‘complete output length’ records the length up to which all strings of that length and below were produced and accounted for in the distributions.

Table 2 shows the counts and percentages of programs in ${\mathrm{IMP}2}_{40}$ according to their halting status. The vast majority of programs are considered non-halting. However, most of them did run to termination but failed to read all the bits in the input stream, meaning they are extensions of a previously executed program corresponding to the same IMP2 sentence but where a smaller input stream, i.e., a prefix, had already been accounted for.

Although the scale of our experiment was very modest, the analysis of the data we were able to collect allowed us to make specific observations in relation to previously suggested properties of CTM complexity approximations.

### 4.1. On the Convergence towards a ‘Natural’ Distribution

In other CTM-related studies, empirical evidence has been reported regarding the correspondence between the CTM complexity rankings produced by different models of computation [9,11,12,13], alluding to an underlying natural distribution of binary strings. In particular, the Spearman correlation coefficient has been employed to assess the correlation between the frequency distributions of binary strings generated by Turing machines, one-dimensional cellular automata, and Post tag systems [9]. This suggests a sort of ‘natural behaviour’, as defined in [12] as “behaviour that is not artificially introduced with the purpose of producing a different-looking initial distribution”. Moreover, it has been suggested that most ‘natural’ models produce similar output frequency distributions [11], indicating some level of empirical invariability in complexity estimations by the CTM, even without a guarantee of optimality.

To test this hypothesis, we believe the IMP2 model is a suitable choice for a reference machine, given its neutrality. While it represents a significant departure from previously chosen ‘natural’ models, it is still reasonable and even optimal. In other words, although the major differences between IMP2 and these other models, which give rise to such conjecture, suggest that IMP2 is unlikely to be biased towards producing a similar output frequency distribution, IMP2 is not designed in an artificial way to produce a different-looking initial distribution. Therefore, we believe this neutral model presents a valuable opportunity to test the hypothesis regarding the convergence towards a ‘natural’ distribution upon application of the CTM.

In our study, the Spearman rank correlation coefficient was also employed to contrast the frequency distribution $D({\mathrm{IMP}2}_{40})$ with the distributions D(4,2) and D(5,2), as published in [5]. To assess the statistical significance of the correlation values, we performed a permutation test and considered *p*-values in the ranges [0,0.001), [0.001,0.01), and [0.01,0.1) to represent very high, high, and low significance, respectively, and 0.1 or larger to represent very low significance.

**Figure 4:**
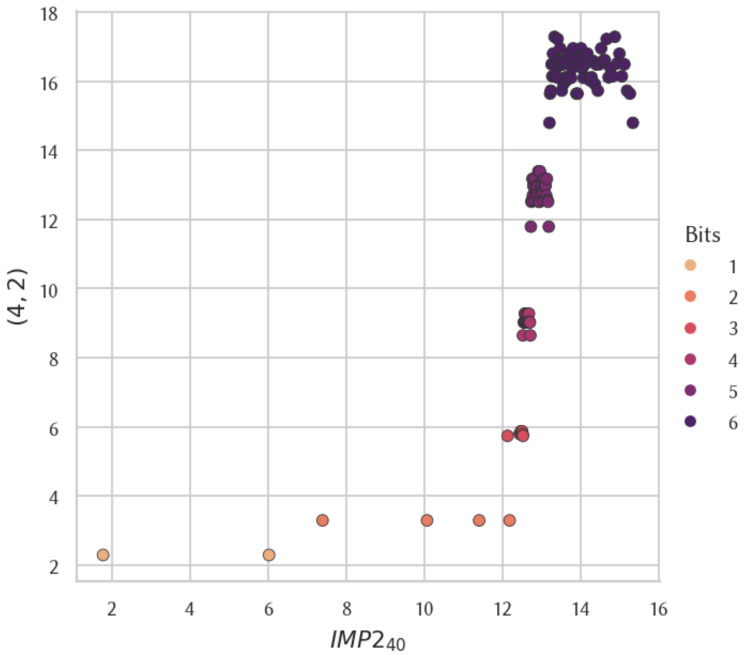
CTM approximation comparison between ${\mathrm{IMP}2}_{40}$ and (4,2).

The results from this study revealed a striking difference when analyzing the data at different resolutions. Globally, when considering all CTM values computed, and locally, by zooming in on the values for strings of fixed lengths, we observed significant variations Upon examining the whole dataset, we found a global correlation of 0.850543 with very high significance between ${\mathrm{IMP}2}_{40}$ and both (4,2) and (5,2) [5,7]. Figure 4 shows the distribution of the approximated CTM values of each string between ${\mathrm{IMP}2}_{40}$ and (4,2). However, when looking at the local Spearman coefficients between ${\mathrm{IMP}2}_{40}$ and both (4,2) and (5,2), we found no correlation, regardless of the length of the strings considered. Altogether, these observations suggest that the global high correlation observed is due to a shared commonality between the three distributions that does not impact the local correlation analysis for strings of a fixed length. This commonality is that in all three distributions, most strings of a given length are less frequent (more complex) than all strings of a smaller length.

While both global and local correlation perspectives provide insights into the compatibility of CTM approximations between reference machines, the discrepancies found suggest that the IMP2 model, though Turing-universal and optimal, might not conform to the natural behaviour defined recently in [14] due to its peculiarities. These include the fact that the model does not display conformance to basic linear transformations (such as binary negation) and that the output distribution produced does not converge to previously reported ‘natural’ distributions and may converge slower to Levin’s universal distribution.

### 4.2. Validation of CTM by SPF

Since our methods for computing the approximations enumerate and execute programs by length in increasing order, it is equally straightforward to count the number of programs producing a string for the CTM as it is to record the length of the smallest program found that produces a string for the SPF. Furthermore, the prefix-free encoding of our IMP2 model yields programs at almost every length, in contrast to previously used models such as Turing machines with *n* states, for which programs are all encoded as strings with $\left\lceil \log_{2}\left({\left(4n+2\right)}^{2n}\right)\right\rceil$ bits [23]. This allows us to run programs across a larger range of lengths. Compared to the CTM, the SPF approximation more closely resembles the original definition of Kolmogorov complexity in Equation (1) and can therefore be considered a suitable approximation, as it serves as a proper upper bound for the actual prefix complexity and provides an alternative measure suitable for validating the corresponding CTM approximation.

**Figure 5:**
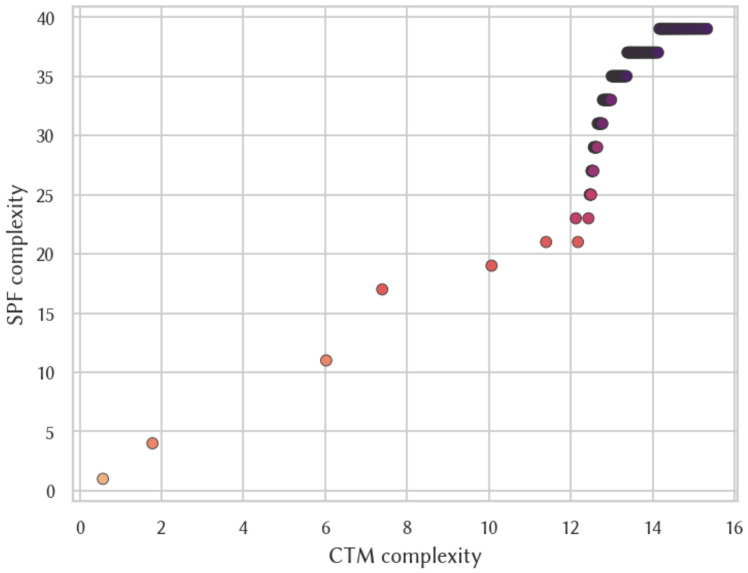
Comparison of complexity estimations for ${\mathrm{IMP}2}_{40}$ for all binary strings of length 6 and below.

**Figure 6:**
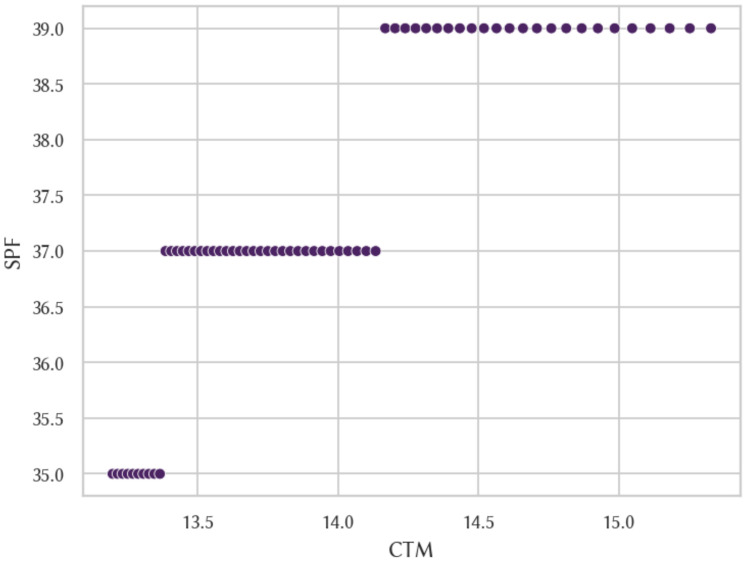
Comparison of complexity approximations for ${\mathrm{IMP}2}_{40}$ for all 6-bit strings.

When contrasting the algorithmic complexity approximated using the CTM and SPF under IMP2 (see Figure 5) we obtain a Spearman correlation coefficient of 0.986114 and a Pearson correlation coefficient of 0.911119, both of which are of very high significance. We also found high and significant correlation coefficients between both measures when looking at strings of particular lengths up to 6 bits (see Figure 6). In other words, we found that both measures were highly correlated from both global and local perspectives. Table 3 details all Spearman correlation coefficients and *p*-values between the frequency distributions produced by the different aforementioned reference machines and also between the CTM and SPF using IMP2.

Although we cannot confidently say that this correlation is not dependent in some way on the chosen reference model, we have no reason to believe that our model is in any way artificially biased in that regard. Therefore, this correlation result comes as a positive surprise since it exceeds theoretical expectations because of the way that the asymptotic term from the Coding Theorem is handled by the CTM. This supports the claim that the CTM is suitable for comparing the relative complexities between output strings. Additionally, our results show that, in most cases, the CTM assigns different complexity values to strings with the same SPF approximation, which may be useful for differentiating strings where the smallest programs found are of the same length. Moreover, a related previous report [15], which involved the execution of a large set of Turing machines with up to five states (and two symbols), showed that the CTM approximation of the complexity of a string agrees with the number of instructions used by the machines producing the string. These considerations give us confidence in using the CTM as a valid substitute for the more naive approach.

## 5. Concluding Remarks

In this paper, we presented a universal optimal model of computation with a simple high-level imperative programming language called IMP2. This model serves as a reference machine for approximating Kolmogorov prefix complexity using the Coding Theorem Method (CTM). In contrast to previous studies that employed low-level models as reference machines, whose optimality is merely conjectured, our IMP2 model was developed with theoretical constraints in mind. This approach enabled us to test previously published hypotheses regarding empirical approximations of complexity using a reasonably constructed reference machine.

The first hypothesis tested was whether CTM approximations would yield similar numerical results when computed with different reference machines, indicating that the approximation might be invariant with respect to the model of computation chosen, when ‘natural’. The observed output frequencies computed using IMP2 strongly aligned with the length-increasing lexicographic ordering of binary strings. While the frequencies obtained with other models show a somewhat similar tendency towards simplicity bias for fixed-length strings, there is little to no correlation. These results suggest that there may be models that are more ‘natural’ than others, even when equally optimal, or not [14]. This implies that IMP2 might require a substantially larger space of programs to observe such convergence. It also remains open whether choices made in the design of IMP2 make it less ‘natural’ and unsuitable for approximating Kolmogorov complexity under the CTM (and other methods). There needs to be further investigation into the sensitivity of the CTM to changes in the choice of prefix-free encoding, enumeration, and input/output conventions, as well as other reasonable high-level models of computation.

The second hypothesis posed was whether the CTM was a valid approach for approximating Kolmogorov complexity. To verify this, we compared the CTM with the SPF, a direct application of the Kolmogorov prefix complexity definition. Our results demonstrate that both approximations are monotonically correlated, meaning there is agreement in the order of assigned complexity values. This indicates that our empirical CTM complexity approximation is stable and meaningful with respect to a fixed reference machine. In addition, there is a practical advantage in choosing the CTM, as it allows for comparing complexities with finer resolution than the SPF. Both approximation methods can be further studied to estimate a lower bound for the constant in the Coding Theorem that is discarded by the CTM.

One of the main challenges we faced in our endeavour was the overwhelming proportion of non-halting programs encountered while executing the experiments with IMP2, resulting in a significant number of executions that did not contribute to the calculation of either the CTM or the SPF. As shown in Table 2, most executions were considered non-halting, and among these, extension programs constituted the majority. It is not straightforward to preemptively omit these subsets of programs from the enumeration or efficiently detect them before running them. This disadvantage is not inherent to the approximation method, as it does not present itself with other reference machines; rather, it is mainly due to the prefix-free code nature of IMP2 programs, which is an essential component for our optimality proof. We posit that there is a trade-off between practicality and optimality when empirically approximating complexity with the present approach.

## Figures and Tables

**Table 1:** Summary of enumerating and executing programs.

	${\mathrm{IMP}2}_{40}$	(4,2)	(5,2)
Total programs	2199020109825	11019960576	26559922791424
Unique strings produced	145	1832	99608
Largest output length	7	16	49
Complete output length	6	8	12

**Table 2:** Halting status for ${\mathrm{IMP}2}_{40}$.

Status	Count	Percentage
Halted	3828109	0.0000017%
Threshold surpassed	887841	0.000040%
Loop reached	392291668910	17.839385%
Read failure	45125277	0.002052%
Extensions	1806678599688	82.158348%

**Table 3:** Spearman correlation coefficients of the frequency of output strings between reference machines (first two columns) and complexity approximations with IMP2. The numbers in parenthesis indicate the correlation significance computed with a permutation test of 20000 samples, given the null hypothesis of a random ranking yielding a higher correlation.

Output Length	IMP2 vs. (4,2)	IMP2 vs. (5,2)	CTM vs. SPF
3	0.0 (0.55622)	0.0 (0.56147)	0.92582 (0.00264)
4	0.0 (0.50062)	0.0 (0.49927)	0.91715 ($4.99975\times \;10^{-5}$)
5	0.0 (0.49837)	0.0 (0.50162)	0.91721 ($4.99975\times \;10^{-5}$)
6	0.0 (0.49832)	0.0 (0.49667)	0.91687 ($4.99975\times \;10^{-5}$)
≤6	0.85052 ($4.99975\times \;10^{-5}$)	0.85052 ($4.99975\times \;10^{-5}$)	0.98577 ($4.99975\times \;10^{-5}$)

## Data Availability

The original data presented in the study are openly available in GitLab at https://kolm-complexity.gitlab.io/optimal-interpreter/ (accessed on 17 September 2024).

## Abbreviations

The following abbreviations are used in this manuscript:

CTM	Coding Theorem Method
SPF	Smallest Program Found
